# Single-Layer Transmissive Chiral Plasma Metasurface with High Circular Polarization Extinction Ratio in Visible Wavelength

**DOI:** 10.3390/nano13050813

**Published:** 2023-02-22

**Authors:** Ran Zhang, Zhichao Zhang, Yuanyi Fan, Hao Zhang, Jinkui Chu

**Affiliations:** 1 Key Laboratory for Micro/Nano Technology and System of Liaoning Province, Dalian University of Technology, Dalian 116024, China; 2 Ningbo Research Institute of Dalian University of Technology, Ningbo 315000, China

**Keywords:** chiral metamaterials, chiral metasurface, circular polarization extinction ratio, circular polarization transmittance difference

## Abstract

Chiral metamaterials are extensively applied in the fields of photoelectric detection, biomedical diagnostics and micro-nano polarization imaging. Currently, single-layer chiral metamaterials are unfortunately limited by several issues, such as a weaker circular polarization extinction ratio and circular polarization transmittance difference. To tackle these issues, a single-layer transmissive chiral plasma metasurface (SCPMs) suitable for visible wavelength is proposed in this paper. Its basic unit is composed of double orthogonal rectangular slots and a spatial π/4 inclined arrangement of the rectangular slot to constitute a chiral structure. Each rectangular slot structure has characteristics that enable the SCPMs to easily achieve a high circular polarization extinction ratio and strong circular polarization transmittance difference. Both the circular polarization extinction ratio and circular polarization transmittance difference of the SCPMs reach over 1000 and 0.28 at a wavelength of 532 nm, respectively. In addition, the SCPMs is fabricated via the thermally evaporated deposition technique and focused ion beam system. This compact structure coupled with a simple process and excellent properties enhances its applicability for the control and detection of polarization, especially during integration with linear polarizers, to achieve the fabrication of a division-of-focal-plane full-Stokes polarimeter.

## 1. Introduction

Chirality, which refers to a property in which a structure cannot be overlapped with its mirror image by translation, rotation or scaling, is a very important concept of molecular structure and a ubiquitous phenomenon in nature [1,2,3,4,5]. A weak optical response is usually exhibited by naturally occurring chiral materials that do not match the wavelength of light on the scale [6,7]. However, this limitation is overcome with artificially designed chiral metamaterials that exhibit a large improvement in optical response and unique optical properties, such as the circular polarization transmittance difference, optical rotation and circular polarization extinction ratio [8,9,10,11]. The circular polarization extinction ratio and circular polarization transmittance difference, which refer to the ratio and differential transmissions of left-handed and right-handed circularly polarized light, respectively, are extensively applied in chemical analysis, molecular detection, biomedical diagnostics and micro-nano polarization imaging [12,13,14,15,16].

Currently, various kinds of chiral metamaterials from three-dimensional (3D) to two-dimensional (2D) can be designed to achieve the circular polarization extinction ratio and circular polarization transmittance difference, the most classical of which is the 3D helical structure. In 2009, a 3D uniform gold-helix chiral structure was proposed by Gansel et al. [17] that combined internal and Bragg resonances, leading to a broadband response with a circular polarization extinction ratio of approximately 10 in the wavelength range of 3.5–6.5 μm and an average circular polarization transmittance difference of over 60%. Since then, various 3D helical structures have been proposed with the aim to broaden the bandwidth and improve the circular polarization extinction ratio and circular polarization transmittance difference, key among them the double-helix, N-helix, hetero-structured double-helix, tapered helix and hybrid helix [18,19,20,21,22,23]. Although the 3D helical structure has superior properties, its main limitation is with regards to the preparation method. The traditional preparation methods are mainly divided into bottom-up and top-down categories. A typical method of the former is the molecular self-assembly method, which is normally utilized to fabricate complex nanostructures [24,25]. The latter method, which includes direct laser writing (DLW), oblique angle deposition (OAD), polystyrene (PS) sphere template technology, electron beam lithography (EBL) and focused ion beam lithography (FIB), is mostly used to manufacture stack layer structures [26,27,28,29,30]. The flexibility of the former relies on DNA, peptides and cysteines as the templates, while the latter is more difficult to use to create structures that work in the visible region due to diffraction limits, as well as the complexity of the fabrication process and the difficulty of controlling the stability of the prepared structures. The combination of various processing methods can compensate to some extent for the shortcomings [31,32,33]. In addition, large-scale fabrication approaches, such as colloidal lithography, nanoimprint lithography and incorporation of molecules into plasmonic metamaterials, are gradually being developed [34]. To relax the usual requirements on fabrication and achieve exotic effects analogous to helix metamaterials, multi-layer stacking structures have been proposed [35,36,37,38,39]. Notably, multi-layer stacking structures are as effective as the 3D helical structures, which achieve a high circular polarization extinction ratio and strong circular polarization transmittance difference due to the coupling between the closely spaced adjacent layers. Although multi-layer stacking structures have appealing properties, they involve the operation of directional alignment and a complicated process, and periodic arrays with sub-wavelength structure and strictly precise layout are still challenging to achieve in the visible wavelength band.

Single-layer chiral metamaterials exhibit asymmetric transmission of circularly polarized light due to the enantiomerically sensitive plasmon excitation and guided mode resonance [40,41,42,43,44,45]. Previous studies show that single-layer non-chiral metamaterials also exhibit strong optical activity and a circular polarization transmittance difference by extrinsic chirality, which mostly relies on the tilted incidence of the light source [46]. Current single-layer chiral metamaterials generally propose a chiral structure directly without giving a specific intuitive design method and process, which mainly explores the effect of each parameter on the structural properties and then seeks the structural parameters with the best performance. Additionally, the current single-layer chiral metamaterials mostly work in the form of reflection and scattering with their response frequencies mainly distributed in the microwave, terahertz wave and near-infrared region [47,48]. To date, compared to the number of chiral metamaterials in wavelengths such as the near infrared, the number of single-layer chiral plasma metamaterials with transmission in the visible wavelength remains relatively few. In 2005, chiral gold nanostructures for visible wavelengths were proposed, which showed that the optical activity in planar structures originated from the 3D nature of the grating and did not violate time-reversal symmetry [49]. In 2021, a metasurface based on rotational symmetric nanoholes was proposed, which achieved effective circular polarization selection in the visible range [50]. To meet the increasing demands for the high performance and efficiency of detectors in the visible band, the circular polarization extinction ratio and circular polarization transmittance difference require further improvement.

In the present study, a single-layer transmissive chiral plasma metasurface (SCPMs) is proposed, which is a composite of double orthogonal rectangular slots and a spatial π/4 inclined arrangement of the rectangular slot to form a chiral structure. The two-part rectangular slot structure fulfills its function, enabling the SCPMs to easily achieve a high circular polarization extinction ratio and strong circular polarization transmittance difference. Summarily, the double orthogonal rectangular slots array and the spatial π/4 inclined arrangement of the rectangular slot array are equivalent to a quarter-wave plate and a linear polarizer, respectively. Notably, the circular polarization extinction ratio and circular polarization transmittance difference of the SCPMs reach over 1000 and 0.28 at a wavelength of 532 nm, respectively. Moreover, the SCPMs exhibits a compact structure with a simple fabrication process and inherits the excellent optical chirality of 3D chiral metamaterials, which are beneficial for applications in detector integration, optical sensing and polarization imaging.

## 2. Structure and Design Methods

**Figure 1:**
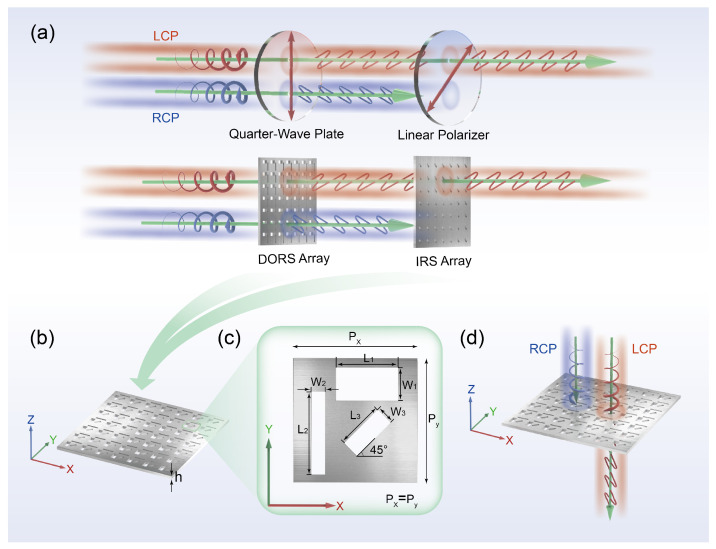
(**a**) Schematic diagram of the single-layer transmissive chiral plasma metasurface (SCPMs). Summarily, the array of double orthogonal rectangular slots (DORS) and the array of spatial π/4 inclined arrangement of the rectangular slot (IRS) are approximately equivalent to a quarter-wave plate and a linear polarizer, respectively. (**b**) Profile of the SCPMs. The aluminum film is etched with an array of periodic structures whose basic unit is composed of DORS and IRS. The thickness h of the aluminum film is 200 nm. (**c**) Unit cell with the associated geometric features. *L*_1_, *W*_1_, *L*_2_, *W*_2_, *L*_3_ and *W*_3_ are set to 258, 136, 338, 56, 198 and 76 nm, respectively. The distance from the center of the spatial π/4 inclined arrangement of the rectangular slot to the center of the double orthogonal rectangular slot is 200 nm. The periods of unit cell are *P_x_* = *P_y_* = 400 nm in the *x* and *y* directions. (We focus on the left-handed structure; if the angle of the tilted rectangular slot becomes −π/4, it will be transformed into a right-handed structure.) (**d**) Illustration of the optical response at the designed wavelength of 532 nm.

Previous studies show that single rectangular slots can exhibit strong transmission resonances, while rectangular slots present strong polarization dependencies and higher transmittance [51,52,53,54]. Therefore, the rectangular slot is chosen as the basic component of the chiral structure array. Figure 1a shows the schematic diagram of the SCPMs. The first half describes the combination of a conventional quarter-wave plate and a linear polarizer, where the fast axis of the quarter-wave plate is oriented at an angle of π/4 to the transmission axis of the line polarizer, which achieves asymmetric transmission of the circular polarization [55,56]. Based on the same principle as above, the second half shows two metasurfaces that are designed for imitation to achieve the same behavior [57,58]. Notably, the SCPMs integrates two metasurfaces with specific various properties on a single plane to form a chiral structure (Figure 1b). Here, we focus on the left-handed structures with the angle of π/4 (the right-handed structures with the angle of -π/4); the optical properties of the left-handed and right-handed structures are opposite. Moreover, its basic unit is given in Figure 1c. A schematic representation of the optical response of the SCPMs at the designed wavelength of 532 nm, indicating different transmittances for left-handed and right-handed circularly polarized light, is shown in Figure 1d.

**Figure 2:**
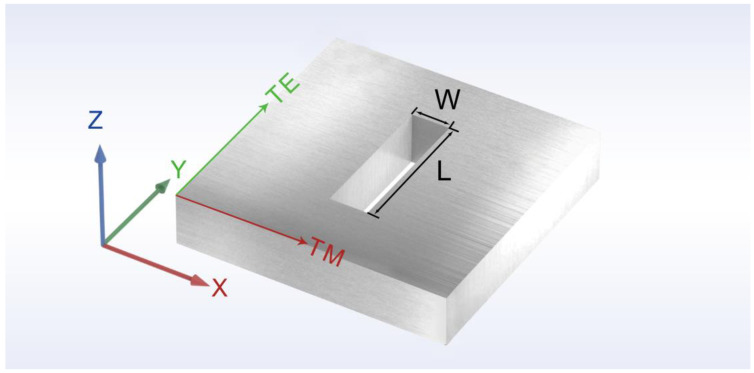
The numerical model of the single rectangular slot. The length and width of a single rectangular slot are *L* and *W*. The thickness of the aluminum film is 200 nm. The electric vector of the TM polarized light was along the *x*-axis, and the electric vector of the TE polarized light was along the *y*-axis.

The structure we proposed is a chiral structure with multiple rectangular slots, and the two-part rectangular slot structure of the basic structural units is optimally designed with the time-domain finite-difference method (FDTD) to achieve the respective properties, aiming to finally realize the high circular polarization extinction ratio and strong circular polarization transmittance difference of the SCPMs. Figure 2 shows the numerical model of the single rectangular slot.

**Figure 3:**
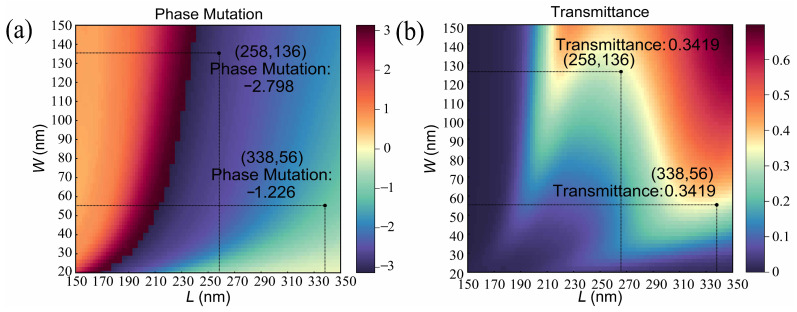
Simulated optical properties of a single rectangular slot. (**a**) Phase mutation of the rectangular slots corresponding to different length (150–350 nm) and width (20–150 nm) ranges at a wavelength of 532 nm and an aluminum film thickness of 200 nm. (**b**) Transmittance of the rectangular slots corresponding to different length (150–350 nm) and width (20–150 nm) ranges at a wavelength of 532 nm and an aluminum film thickness of 200 nm.

During the 3D simulation, the linear polarized light with a wavelength of 532 nm is incident along the positive direction of the *z*-axis. The electric vector of the TM polarized light is along the *x*-axis, and the electric vector of the TE polarized light is along the *y*-axis. A monitor is set up at a suitable location in the *z*-axis direction for collecting information, such as the transmittance and phase. The simulation unit is enclosed by periodic boundary conditions in both the *x*-axis and *y*-axis directions. Perfectly matched layers (PMLs) are employed along the *z*-axis direction. The accuracy of the mesh is 1 × 1 × 20 nm. The simulation period is set to 400 × 400 nm. The refractive indexes of aluminum given by Palik [59] are used in the simulations. The simulation model does not include the substrate. Considering the structure period and the need to prevent mutual interference in the geometric dimensions between the rectangular slots, the length of the rectangular slots is set to scan in the range of 150–350 nm, and the width of the rectangular slots is set to scan in the range of 20–150 nm. Figure 3a,b shows the phase mutation and transmittance corresponding to different sizes of the rectangular slots at a wavelength of 532 nm and an aluminum film of 200 nm.

**Figure 4:**
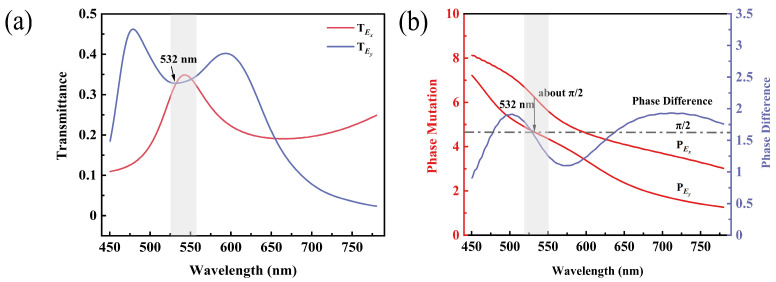
Simulated optical properties of the double orthogonal rectangular slots array. (**a**) The transmittance of the TM and TE linear polarized light (*T_Ex_* and *T_Ey_*) of the double orthogonal rectangular slots array. (**b**) The phase mutation of the TM and TE linear polarized light (*P_Ex_* and *P_Ey_*) of the double orthogonal rectangular slots array.

The Jones matrix of the quarter-wave plate with zero azimuth is:

(1) $$T_{1}=\left[\begin{array}{cc} \exp\left(\frac{i\pi }{4}\right) & 0\\ 0 & \exp\left(-\frac{i\pi }{4}\right) \end{array}\right],$$

where the two elements of the subdiagonal are 0, indicating that there is no linear polarization transition effect. The two elements of the main diagonal need to satisfy equal amplitudes and a phase difference of π/2. When double orthogonal rectangular slots have the dimensions *L*_1_ = 258 nm, *W*_1_ = 136 nm, *L*_2_ = 338 nm and *W*_2_ = 56 nm, they realize the same transmittance and phase difference of π/2. Subsequently, the property of the double orthogonal rectangular slots array at this size is verified with the time-domain finite-difference method (FDTD). Figure 4a,b shows the transmittance and phase mutation of the TM and TE linear polarized light of the double orthogonal rectangular slots array. The bandwidth of the TM and TE linear polarized light with a transmittance difference within 10% is 526–558 nm, and the bandwidth of the TM and TE linear polarized light with a phase difference of π/2 within 10% is 523–540 nm. Especially at the target wavelength of 532 nm, the transmittance is the same and the phase difference is approximately π/2, so the double orthogonal rectangular slots array at this size realizes the performance of a quarter-wave plate. Meanwhile, the simulation results also demonstrate that the coupling between the double orthogonal rectangular slots has a small effect on its performance.

**Figure 5:**
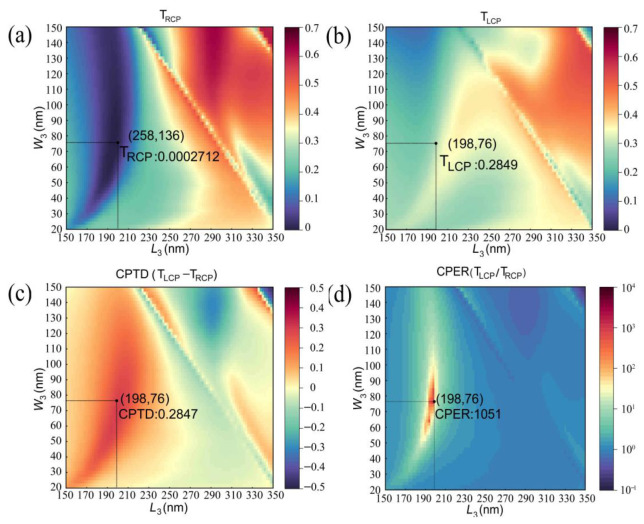
Simulated optical properties of the composite structures. (**a**) The transmittance of the right-handed circularly polarized light (T_RCP_). (**b**) The transmittance of the left-handed circularly polarized light (T_LCP_). (**c**) Circular polarization transmittance difference (CPTD). (**d**) Circular polarization extinction ratio (CPER).

Finally, a composite simulation of the size-determined double orthogonal rectangular slots and spatial π/4 inclined arrangement of the rectangular slot is performed with the length and width of the spatial π/4 inclined arrangement of the rectangular slot scanned in the same range. During the 3D simulation, the *x*-axis and *y*-axis directions are set as the periodic boundary condition, and the *z*-axis direction is the perfect match layer boundary condition. The excitation sources are two linear polarized light sources with a ±π/2 phase difference perpendicular to each other, which are constructed into right-handed and left-handed circularly polarized light (RCP and LCP). The handedness of the circularly polarized light is defined from the point of view of the receiver. The right-handedness corresponds to a clockwise rotation, and the left-handedness corresponds to an anti-clockwise rotation. The simulation period is set to 400 × 400 nm, and the accuracy of the mesh is 1 × 1 × 20 nm. The refractive indexes of aluminum are the same as that set in the previous simulation. Some of the more detailed parameters of the aluminium are as follows: the plasma frequency ω*_p_* is at 15.05 eV [60], and the full width at half-maximum Δ*E*_1/2_ of the electron–energy–loss function is at 0.54 eV [61]. As with the previous simulation, the substrate is not included here. Figure 5a,b shows the transmittance of the right-handed and left-handed circularly polarized light (T_RCP_ and T_LCP_). The circular polarization transmittance difference is denoted as CPTD = T_LCP_ − T_RCP_, whereas the circular polarization extinction ratio is represented by CPER = T_LCP_/T_RCP_ (Figure 5c,d). When the length (*L*_3_) and width (*W*_3_) of the spatial π/4 inclined arrangement of the rectangular slot are 198 nm and 76 nm, respectively, the circular polarization extinction ratio of the SCPMs reaches a maximum.

**Figure 6:**
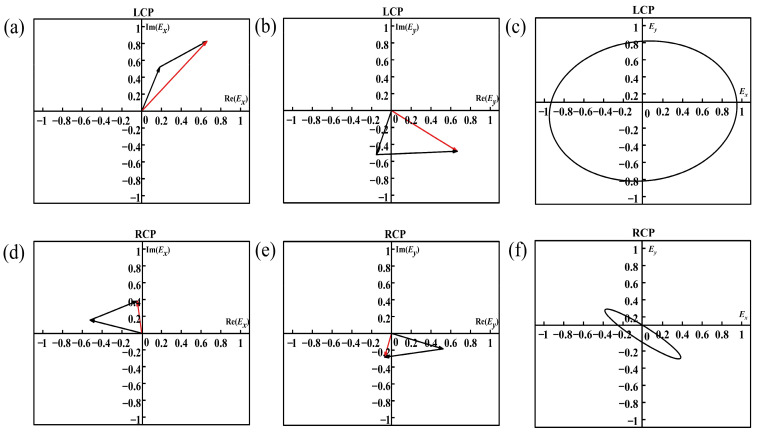
Simulation of design ideas. (**a**,**b**) The two black vectors represent the left-handed circularly polarized light passing through the double orthogonal rectangular slots array and the spatially π/4 tilted arrangement of the rectangular slot array, and their respective components of the emitted light in the *x* and *y* directions; the red vectors represent their sum vectors. (**c**) The *Ey* and *Ex* at different times (under the left-handed circularly polarized light) (**d**,**e**) The two black vectors represent the right-handed circularly polarized light passing through the double orthogonal rectangular slots array and the spatially π/4 tilted arrangement of the rectangular slot array, and their respective components of the emitted light in the *x* and *y* directions; the red vectors represent their sum vectors. (**f**) The *Ey* and *Ex* at different times (under the right-handed circularly polarized light).

The proposed structure is composed of tightly packed double orthogonal rectangular slots that are designed to induce a phase difference of π/2 on the *x* and *y* components as well as a spatial π/4 inclined arrangement of the rectangular slot that filters out only a single linear polarization after the double orthogonal rectangular slots. As shown in the simulation results of Figure 5, the high circular polarization extinction ratio and strong circular polarization transmittance difference are achieved. This phenomenon is the result of our adjustment of the geometry of the spatial π/4 inclined arrangement of the rectangular slot to achieve the enhancement and weakening of the interference of the electric field. The SCPMs we designed is split into two separate array structures: the array structure of a double orthogonal rectangular slot and the array structure of a spatial π/4 inclined arrangement of the rectangular slot. The two separate array structures are each simulated separately with the incident light from both structures being the same left-handed and right-handed circularly polarized light at a wavelength of 532 nm. Under the illumination of light with the same spin direction, the outgoing light from the two structures is vector superimposed in the *x* and *y* directions. The electric field vector diagram is given in Figure 6. Figure 6a,b shows that the left-handed circularly polarized light passes through the double orthogonal rectangular slots array and spatial π/4 inclined arrangement of the rectangular slot array, and the components of their respective outgoing light in the *x* and *y* directions are coherently enhanced. The right-handed circularly polarized light passes through the double orthogonal rectangular slots array and spatial π/4 inclined arrangement of the rectangular slot array, and the component coherence of their respective outgoing light in the *x* and *y* directions is coherently weakened (Figure 6c,d). Although there is a coupling effect between multiple rectangular slots, the overall trend is consistent, which proves the reasonableness and correctness of our design method.

**Figure 7:**
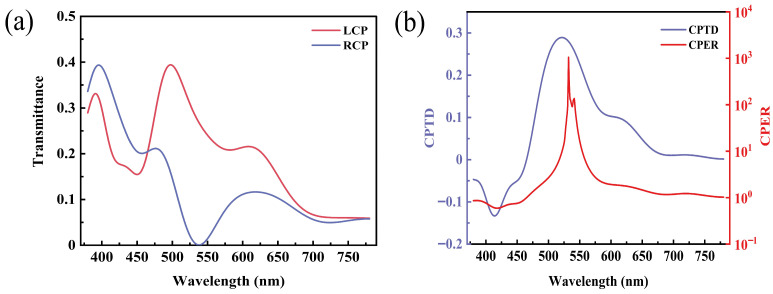
Simulation optical properties of the SCPMs. (**a**) Transmittance of the left-handed and right-handed circularly polarized light (T_LCP_ and T_RCP_) by the full visible band (380–780 nm) simulation. (**b**) Circular polarization transmittance difference (CPTD = T_LCP_ − T_RCP_) and circular polarization extinction ratio (CPER = T_LCP_/T_RCP_) obtained by the T_RCP_ and T_LCP_.

To verify the performance of the SCPMs at the above-mentioned dimensions, simulations for the full visible band are performed. The transmittances of the right-handed and left-handed circularly polarized light are given in Figure 7a. The circular polarization transmittance difference and circular polarization extinction ratio are given in Figure 7b. Briefly, the circular polarization transmittance difference of the SCPMs is greater than 0.1 in the wavelength range of 478–609 nm, whereas the circular polarization transmittance difference reaches 0.28 at a wavelength of 532 nm. Moreover, the circular polarization extinction ratio is greater than 10 in the wavelength range of 521–553 nm and reaches approximately 1000 at a wavelength of 532 nm.

## 3. Device Fabrication and Characterization

### 3.1. Device Fabrication

**Figure 8:**
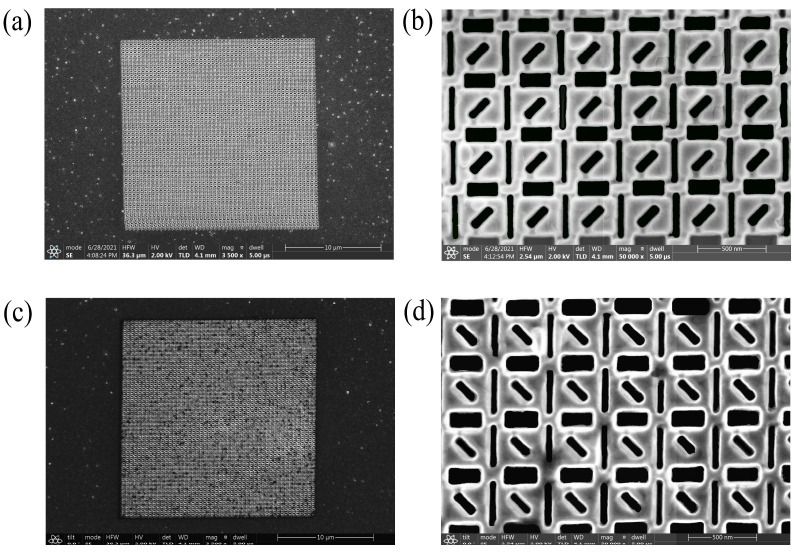
The SEM images of the fabricated samples. (**a**) The SEM images of the left-handed structure. (**b**) The SEM partial images of the left-handed structure. (**c**) The SEM images of the right-handed structure. (**d**) The SEM partial images of the right-handed structure.

Focused ion beam (FIB) is utilized to process micro and nano structures and uses a focused beam of ions (e.g., gallium, helium, neon) to sputter atoms away from a sample surface to form a desired nanostructure. For the structural form of the SCPMs, the sample is fabricated with the thermally evaporated deposition technique and focused ion beam system. First, the SiO_2_ substrate is pretreated with operations such as cleaning. Then, a 200 nm-thick aluminum film is deposited on a pretreated SiO_2_ substrate using the thermally evaporated deposition technique. Finally, the designed chiral patterns are milled in the aluminum film using the focused ion beam system. The size of the fabricated structure array area is 20 × 20 µm. There are two main process parameters for focused ion beam: voltage (30 Kv) and ion beam current (41 pa), where the ion beam current corresponds to the beam spot diameter. The geometric features of the samples are characterized with scanning electron microscopy (SEM). Figure 8a,c shows the SEM images of the left-handed and right-handed structures. Figure 8b,d shows the SEM partial images of the left-handed and right-handed structures.

### 3.2. Performance Testing

**Figure 9:**
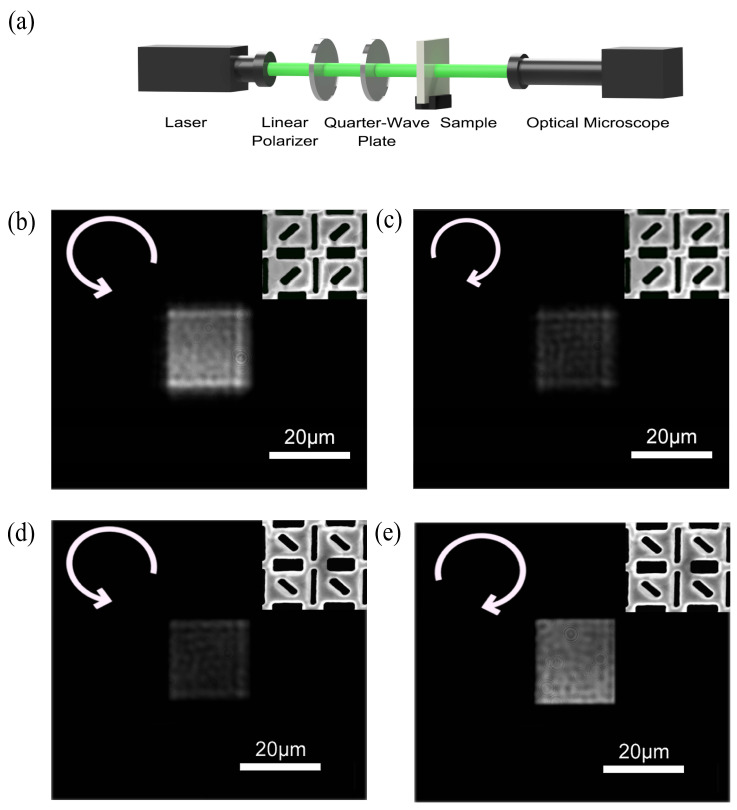
Performance testing. (**a**) Experimental setup for performance verification of the SCPMs. (**b**,**c**) Grayscale images of the sample of the left-handed structures under the left-handed and right-handed circularly polarized light. (**d**,**e**) Grayscale images of the sample of the right-handed structures under the left-handed and right-handed circularly polarized light. The handedness of the circularly polarized light is defined from the view of the receiver (right-handedness corresponds to a clockwise rotation, and left-handedness corresponds to an anti-clockwise rotation).

Experimental test instrumentations are set up to test the optical properties of the fabricated samples (Figure 9a). In the experiment, the linear polarized light with a wavelength of 532 nm emitted by the laser is converted to circularly polarized light by combining a linear polarizer and a quarter-wave plate. Here, the fast axis of the quarter-wave plate is at an angle of ±π/4 to the polarization direction of the linear polarizer and then irradiated normally onto the sample. Next, the transmission intensity distribution of the sample is collected using an optical microscope followed by the acquisition of grayscale images of the SCPMs under left-handed and right-handed circularly polarized light illumination. Figure 9b,c shows the grayscale images of the sample of the left-handed structures under the left-handed and right-handed circularly polarized light. Figure 9d,e shows the grayscale images of the sample of the right-handed structures under the left-handed and right-handed circularly polarized light.

**Figure 10:**
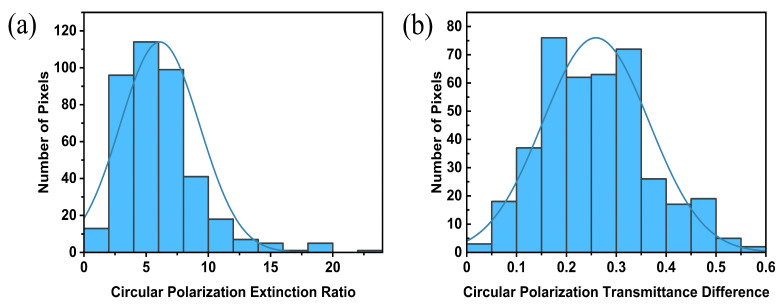
Histograms showing the distribution of (**a**) the circular polarization extinction ratio (CPER) and (**b**) the circular polarization transmittance difference (CPTD) across 400 regions.

First, the target area of the experimentally acquired images are divided into 400 regions according to 10 × 10 pixels. Then, the circular polarization extinction ratio and circular polarization transmittance difference of the corresponding regions are counted. The normal distributions of the circular polarization extinction ratio and circular polarization transmittance difference across 400 regions are given in Figure 10a,b. The horizontal coordinates correspond to the peaks of the normal distribution representing the average values of the circular polarization transmittance difference and circular polarization extinction ratio, which reached 6.02 and 0.25, respectively. We extract pixel values of the sample grayscale image corresponding to 80% of the area and obtain a result in which the CPER of the SCPMs reaches 6.

Table 1 shows a comparison of the most important parameters of the SCPMs we proposed with other chiral metastructures. The 3D helical structures and multi-layer stacking structures operate mostly in the near-infrared band. They offer superior performance but are difficult to fabricate. Compared to the 3D helical structures and multi-layer stacking structures, the production process of a single-layer flat structure is simple. The SCPMs we proposed is suitable for visible wavelengths. Both the circular polarization extinction ratio and circular polarization transmittance difference of the SCPMs reach over 1000 and 0.28 at a wavelength of 532 nm, respectively. After experimental testing, the CPTD and CPER of the sample reach 0.22 and 6 at a wavelength of 532 nm, respectively.

**Figure 11:**
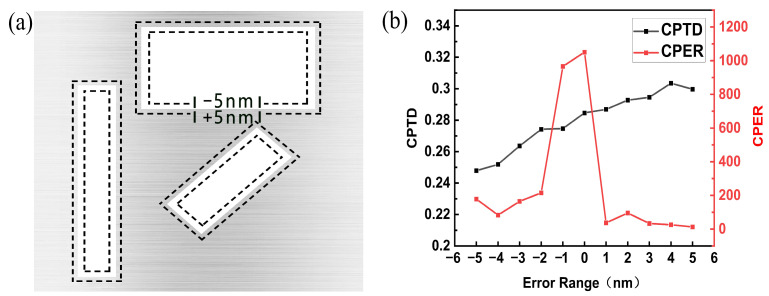
(**a**) Schematic diagram of the basic cell structure with the ±5 nm dimensional error range. (**b**) Simulated optical properties of the SCPMs with the dimensional error of the rectangular slots.

The experimental and simulated results do not correspond well, mainly related to two reasons. On the one hand, this may have resulted from a sample fabrication error, including the dimensional error of the rectangular slots and the surface topography error of the aluminum film. The processing error range of the focused ion beam system used to fabricate the sample is ±5 nm; a schematic representation of the basic cell structure within the ±5 nm dimensional error range is shown in Figure 11a. Figure 11b shows the simulated optical properties of the SCPMs with the dimensional error of the rectangular slots. As can be observed, the dimensional error of the rectangular slots has a significant impact on the performance of the SCPMs. The circular polarization transmittance difference and circular polarization extinction ratio of the SCPMs are reduced by an average of 2 and 82.7%, respectively, with the lowest circular polarization transmittance difference and circular polarization extinction ratio reaching 0.24 and 13, respectively.

On the other hand, the differences between the experiments and simulations may be attributed to testing error, including the angle error of the quarter-wave plate and linear polarizer and the phase-delay error of the quarter-wave plate. The schematic diagram of the experimental test instrumentations that tested the optical properties of the fabricated samples is shown in Figure 9a, which contains a laser light source, a linear polarizer, a quarter-wave plate, the sample and an optical microscope. The Stokes vector can be used to characterize light in any of its polarization states. In this case, natural light is represented by Equation (2), the laser and the linear polarizer are used to produce linear polarized light.

(2) $$s_{in}=\left[\begin{array}{c} I\\ 0\\ 0\\ 0 \end{array}\right],$$

where *I* denotes the light intensity of the light source. The linear polarizer (WP25M-VIS) used in the experiment was purchased from Thorlabs. The working wavelength ranges from 420 nm to 700 nm, and the average extinction ratio is approximately 800:1. Because of the large extinction ratio of the chosen linear polarizer, it can be regarded as an ideal linear polarizer. The optical behaviors of the linear polarizer with an azimuth of zero degrees can be represented by the Mueller matrix

(3) $$P_{1}=\frac{1}{2}\left[\begin{array}{cccc} 1 & 1 & 0 & 0\\ 1 & 1 & 0 & 0\\ 0 & 0 & 0 & 0\\ 0 & 0 & 0 & 0 \end{array}\right].$$

The quarter-wave plate (AQWP05M-580) was purchased from Thorlabs. The Mueller matrix of the quarter-wave plate can be expressed as

(4) $$P_{2}=\left[\begin{array}{cccc} 1 & 0 & 0 & 0\\ 0 & 1-\left(1-\cos\alpha \right)\sin^{2}2\theta & \left(1-\cos\alpha \right)\sin2\theta \cos2\theta & -\sin\alpha \sin2\theta \\ 0 & \left(1-\cos\alpha \right)\sin2\theta \cos2\theta & 1-\left(1-\cos\alpha \right)\cos^{2}2\theta & \sin\alpha \cos2\theta \\ 0 & \sin\alpha \sin2\theta & -\sin\alpha \cos2\theta & \cos\alpha \end{array}\right],$$

where *θ* is the relative angle between the fast axis of the quarter-wave plate and the polarization direction of the linear polarizer, and *α* is the phase delay of the quarter-wave plate. The Mueller matrix of a sample can be presented as

(5) $$P_{3}=\frac{1}{2}\left[\begin{array}{cccc} 1 & 0 & 0 & 1\\ 0 & 0 & 0 & 0\\ 0 & 0 & 0 & 0\\ 1 & 0 & 0 & 1 \end{array}\right].$$

The final Stokes vector of the output light can be obtained with the following function:

(6) $$S_{out}=P_{3}P_{2}P_{1}S_{in}.$$

The first element of the Stokes vector represents the light intensity. Then, the light intensity grayscale response detected by the detector is

(7) $$S_{out-i}\left(1,1\right)=\frac{KI}{4}\left(1+\sin\alpha \sin2\theta \right),$$

where *K* represents the quantum efficiency of the detector. The circular polarization extinction ratio refers to the ratio of transmissions of the left-handed and right-handed circularly polarized light. The following calculation is implemented:

(8) $$\frac{S_{out-}{}_{1}\left(1,1\right)}{S_{out-}{}_{2}\left(1,1\right)}=\frac{1+\sin\alpha_{1}\sin2\theta_{1}}{1+\sin\alpha_{2}\sin2\theta_{2}}.$$

**Figure 12:**
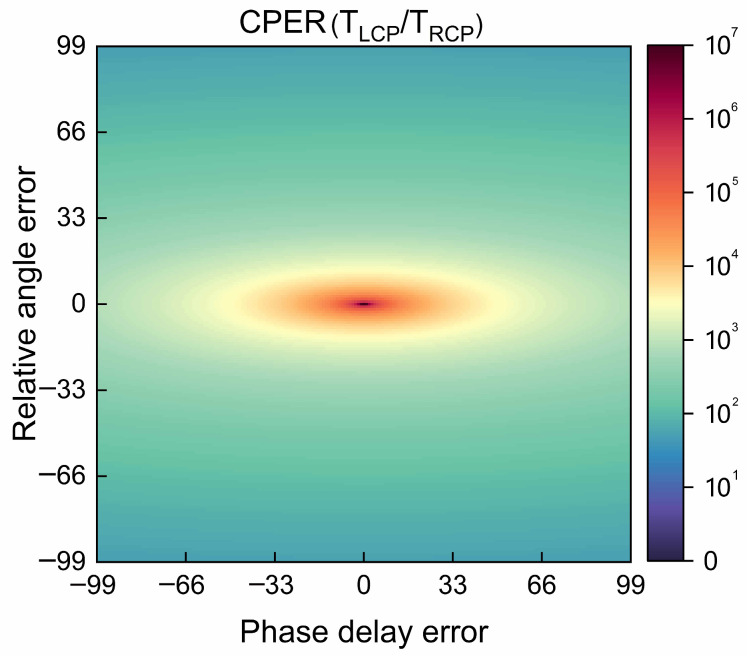
Effects of the relative angle error and phase-delay error on the measurement results of the circular polarization extinction ratio.

The relative angle error is ±0.035 rad, and the phase-delay error is ±0.27 rad. Figure 12 shows the effect of the relative angle error and phase-delay error on the measurement results of the circular polarization extinction ratio, and the lowest circular polarization extinction ratio is 50.08. As can be observed, the relative angle error and the phase-delay error have a significant impact on the performance of the SCPMs.

In addition, there is a possibility that material loss deteriorates the performance of the device. In short, the inconsistency between the experimental and simulation results is the result of a combination of factors. To reduce the dimensional processing error in the rectangular slot, we can use the magnetron sputtering method when preparing the aluminum film and optimizing the FIB parameters, etc. In addition, the test error can be reduced with multiple measurements.

## 4. Conclusions

In this paper, a single-layer transmissivity chiral plasma metasurface (SCPMs) suitable for visible wavelength is proposed to achieve a high circular polarization extinction ratio and strong circular polarization transmittance difference. The structure of the proposed SCPMs is based on that of the double orthogonal rectangular slots array and spatial π/4 inclined arrangement of the rectangular slot array. Compared to the 3D helical structures and multi-layer stacking structures, the fabrication process of the SCPMs is much simpler. The SCPMs has a high circular polarization extinction ratio and strong circular polarization transmittance difference in the visible light region. Specifically, the average circular polarization extinction ratio and circular polarization transmittance difference are greater than 30 and 0.2, respectively, in the 500–600 nm simulation band. The maximum circular polarization extinction ratio and circular polarization transmittance difference reach over 1000 at a wavelength of 532 nm and 0.28 at a wavelength of 521 nm, respectively. In addition, the sample is prepared with thermal vapor deposition and FIB, and the test results demonstrate an obvious circular polarization transmittance difference and circular polarization extinction ratio. Notably, its compact and novel structural design coupled with ultra-thin thickness, simple process, and excellent performance make it a promising candidate for exciting applications for polarization control, especially because it has great potential as a circular polarizer to integrate with a linear polarizer for real-time full-Stokes polarization detection. Moreover, the design method of this structure can also be used for the design of other waveband devices. Finally, the main focus for the future of single-layer chiral materials should be to break the limitations of narrow frequency response to expand the operating bandwidth.

## Figures and Tables

**Table 1:** Comparison of chiral metamaterials.

Chiral Metamaterials		CPTD	CPER	Operating Wavelength or Frequency	Manufacturing Scalability
3D helical structures	Helix [17]	>0.6 (simulation) ~0.6 (experiment)	~10 (simulation) ~9 (experiment)	3.5–6.5 μm	Challenging: complex 3D fabrication
	Tapered helix [22]	~0.4 (simulation) ~0.35 (experiment)	>10 (simulation) ~10 (experiment)	30–90 THz	
multi-layer stacking structures	Twisted-Arc [37]	~0.35 (simulation) ~0.35 (experiment)	<10 (simulation) <10 (experiment)	1.2–1.5 μm	Challenging: sensitive to alignment between multilayers
	Layer-to-layer connection [39]	~0.7 (simulation)	6.9 (simulation)	4.69–8.89 μm	
2D	Full media z-through hole [45]	~0.7 (simulation) ~0.6 (experiment)	NA	1.50–1.61 μm	Yes
	Single Spiral [40]	NA	~100 (simulation)	808 nm	
	Double spiral [41]	NA	<10 (simulation)	695 nm	
	Rotational symmetric nanoholes [50]	~0.1 (simulation)	NA	633 nm	
	This work	0.28 (simulation) 0.22 (experiment)	1051 (simulation) 6 (experiment)	532 nm	

## Data Availability

Data underlying the results presented in this paper are not publicly available at this time but may be obtained from the authors upon reasonable request.
